# Supplementary material for: A Notch-dependent transcriptional mechanism controls expression of temporal patterning factors in Drosophila medulla
Source: eLife. 2022 Aug 30;11:e75879. doi: 10.7554/eLife.75879 (PMC9427115; doi:10.7554/eLife.75879)
Supplement: Supplementary file 1. [file elife-75879-supp1.docx]

Sequence of fragment deleted by CRISPR-Cas9 targeting the u8772 220bp enhancer (5’ -> 3’)

2L: 3817278-3817675

cgcctcgcccaaatgcatttgaatcgagtggtgagcgatagttttcacatgttttgttcgagcattaattccgtttaaatgatttccgcaacagatttgagctttgcacgtaatcaagtggaaatgaatttgaccaacacaagaggtttatacacacatcacattttctgcctttatgtttttgcggtgtcccattagtttgattgtttcgaaggccactgagccgtgaaaaatatcagagaaataaaaatcgaaataccgaaatgagctggttttttgttagcgaaagtgcagatttttcaggactcgcaaagggatgtgattgaagatcttcaggatatttcagcacatgcatcgatatttgtcccaaactggaagtcattgacccagttactttt

Sequence of fragment deleted by CRISPR-Cas9 targeting the d5778 850bp enhancer

2L: 3842548-3844867

aagtccttgggtaatacgaggagaacaaaaacaagtaaggataatattttcataggcaaaacaattgtggttgtgcaaggaagtgacttttgggaacgggaggcacttgcagctgcccaagtatatccaccatatcccatatcctttttccgagtccctaatttcctgggaaagctttcggtgcagttcgccagccaaaacacttgagcacttaaaaaggcgcattaactcgagtctggtttccgattccgatttcgcttcctcctgccaacttatttctatatcttctccctttgtgccctgtgtgtgtgaaacaaaaacgtttgtttcaatacgttggcttcgtgcattttacggtgttgggaaacagacgaaatggactcattgattccaattgactgatttcaattgatgttaagtgtctgccacagtcgcagccgcaaattcagtggcacaactccgtcgcagccaaatgccatttgcttttcacatccaggtcgaacggcgttgccttgttgactttgtttttgctactcattgccgcgatttgggttaggcatggggtatgtgcgcactgtgggaactttggattactcagatgaaacagcatttaggacactatgcagctggaaagataaactagttgatagctactcatttactcatttactacttactactaatttaatgcatttttaacaactttaagctacacaagccaaaactaatgggtattttatagtcctatttaacccctttaacgaatgcatccttttaccttttttggtcacggcagctgaactctgccctttcgttgggggtgactcctccctcccgtactccctccctccctcccctccctctccgcgccacagtcgaccttgtcaagtaccttgttagctgttgggcaaatgtgccacacaagtggctcacatcagcgggatcgaaaataaaaagcgaaacgcatcgagaacttcccaagaaaacggcgagtcaaagttgagaaaacgctgcttccgtttaattgacaattgaacccgaacccggaccgaactcctggagaatatgtacgctgctatccggcatagtcgagtcatcccaagtcatgcgcttaatttcccgtttaaaacgccattcattcaattaagcgaatgaatttgtggcacggcagacgacagcagaagtttttttttcctgaaagaagactatcatcattgagatgccccgagatctctcggctggagctccagatccgatgcgatccaatcggatgagatgagatcgtatggaataggatggagcatgcgggtctctggggtctgtggtctttcgatctttttgtctgcccccgggggctttcttcagtaatcagacgcgggtcaaaatatttaccacttgaccctattgatttaattaaatagtttcaagacattcaactgtttgttggccgcgcaaaatgaatttcgtgtgcgagataactcagatacagtagctgcagtagctgatcaactatctttcagatatgcgcacatttctctctcgttttgtctccgagctgtcaacacagattccaaactgcagacgtgttaattaacgacagagttaactaattgttgttagcaaaatattttcgtaattcgatactaaattcgagttccgctcaacttgcttgcttgtgggcttttgtgttgaacagcacaagacctcaaggaacagtggaacagtgcttcaaagtggggaaaaacatcttataatctagatcatttttaaatttcataaagtctttgattgaaataaaacgtttaagtgaggcaagttcgtattattattagagaaagataactaacctttttggtttatatttaggtcttgaagcactgttcctgctgttttcgctgttgactgcccatgaaacgcttaactgcgagtggcaattggatacctggctgaatccaaatacgaatcttaatctgaatctgcgagtctttgtggccaatgaacacggcagcggcacaacacaaaaacttagcagatactcatgttttatttgtgcatttcgcgcgcgcgttccacttggaaatgctctgtggcaccaaaaggagccactgatgtccaaaatcaaaatggtttcagttcgccggggcaatgcctaaaatgtttctttgtttttgtgttccctgttggcaagcggcgcttcaacagatacagatagatgtgaaatttgttgctaaaaaaaaaagtaagtccataaatcaaatgcctttaaatattcatgagtcaggacaatgtgtgaacaagcgaaaggagctgggatatcgaa
