# Supplementary material for: A Notch-dependent transcriptional mechanism controls expression of temporal patterning factors in Drosophila medulla
Source: eLife. 2022 Aug 30;11:e75879. doi: 10.7554/eLife.75879 (PMC9427115; doi:10.7554/eLife.75879)
Supplement: Supplementary file 4. [file elife-75879-supp4.docx]

**Supplementary Table 3.** **List of gene blocks used for reporter assays and for cloning Ey coding sequence into the pUAST-Dam-attB plasmid**. Restriction enzyme sites (underlined) and a few overhang bases are added to facilitate cloning.

| 220-Ey-1d (199 bp) |
| --- |
| TTAGGCGCGCCGAATCGAGTGGTGAGCGATAGTTTTCACATGTTTTGTTCGAGCATCGCAACAGATTTGAGCTTTGCACGTAATCAAGTGGAAATGAATTTGACCAACACAAGAGGTTTATACACACATCACATTTTCTGCCTTTATGTTTTTGCGGTGTCCCATTAGTTTGATTGTTTCGAAGGCCACTGAGCCGTGAAAAATATCAGAGCGGCCGCATATT |
| 220-Ey-2m (220 bp) |
| TTAGGCGCGCCGAATCGAGTGGTGAGCGATAGTTTTCACATGTTTTGTTCGAGCATTAATTCCGTTTAAATGATTTCCGCAACAGATTTGAGCTTTGCACGTAATCAAGTGGAAATGAATTTGACCAACACAAGAGGTTTATACACACATCACATTTTCTGCCTTTATGTTTTTGCGGTGTCCCATTAGTTTGATTGTTTCGAAGGCCACATAGCCGTGAAAAATATCAGAGCGGCCGCATATAT |
| 220-SuH-1-4m (220 bp) |
| TTAGGCGCGCCGAATCGAGTGGTGAGCGACTAGGAGACGATGTTTTGTTCGAGCATTAATTCCGTTTAAATGATTTCCGCAACAGATTTGAGCTTAGACTGTAATCAAGCGTAAGTGGATTTGACCAACACAAGAGGTTTATACACACATCACATTTTCTGCCTTTATGTTTTTGCGGTGTCCCATTAGTTTGATTGTTTCGAAGGCCACTGAGCCGTAAAGCGAGTCAGAGCGGCCGCATATAT |
| 220-SuH-1m4m (220 bp) |
| TTAGGCGCGCCGAATCGAGTGGTGAGCGACTAGGAGACGATGTTTTGTTCGAGCATTAATTCCGTTTAAATGATTTCCGCAACAGATTTGAGCTTTGCACGTAATCAAGTGGAAATGAATTTGACCAACACAAGAGGTTTATACACAATCACATTTTCTGCCTTTATGTTTTTGCGGTGTCCCATTAGTTTGATTGTTTCGAAGGCCACTGAGCCGTAAAGCGAGTCAGAGCGGCCGCATATAT |
| 220-Scro-1m (220 bp) |
| TTAGGCGCGCCGAATCGAGTGGTGAGCGATAGTTTTCACATGTTTTGTTCGAGCATTAATTCCGTTTAAATGATTTCCGCAACAGATTTGAGCTTTGCACGTAATCGTGTGGAAATGAATTTGACCAACACAAGAGGTTTATACACACATCACATTTTCTGCCTTTATGTTTTTGCGGTGTCCCATTAGTTTGATTGTTTCGAAGGCCACTGAGCCGTGAAAAATATCAGAGCGGCCGCATATAT |
| 220-Slp1-1m2m (220 bp) |
| TTAGGCGCGCCGAATCGAGTGGTGAGCGATAGTTTTCACATGTACCAAGCGAGCATTAATTCCGTTTAAATGATTTCCGCAACAGATTTGAGCTTTGCACGTAATCAAGTGGAAATGAATTGATCGACTCCAAGAGGTTTATACACACATCACATTTTCTGCCTTTATGTTTTTGCGGTGTCCCATTAGTTTGATTGTTTCGAAGGCCACTGAGCCGTGAAAAATATCAGAGCGGCCGCATATAT |
| Ey gene block for cloning into pUAST-attB-Dam plasmid |
| AGATCTGCGGCCGCAAATGTTTACATTGCAACCAACTCCAACTGCTATAGGCACCGTGGTTCCCC  CATGGTCAGCGGGAACATTGATAGAGCGCCTGCCGTCTTTAGAAGACATGGCTCACAAGGGTCACAGTGGAGTAAATCAGCTGGGTGGCGTTTTTGTTGGAGGAAGGCCTTTGCCAGATTCAACACGGCAAAAAATTGTCGAACTGGCACATTCTGGAGCTCGGCCATGTGATATTTCTCGAATTCTGCAAGTATCAAATGGATGTGTGAGCAAAATTCTCGGGAGGTATTATGAAACAGGAAGCATACGACCACGTGCTATCGGAGGATCCAAGCCACGTGTGGCCACAGCCGAAGTCGTTAGCAAAATTTCGCAGTACAAACGCGAGTGTCCTAGCATATTTGCTTGGGAAATTCGGGATAGATTACTTCAGGAGAACGTTTGTACTAACGATAATATACCAAGTGTGTCCTCAATAAACCGTGTATTGAGAAACTTGGCTGCGCAAAAGGAGCAGCAAAGCACGGGATCCGGGAGCTCCAGCACATCCGCCGGCAACTCAATCAGCGCAAAAGTGTCTGTCAGCATCGGTGGCAACGTGAGCAATGTGGCAAGCGGATCGAGAGGCACGTTGAGCTCTTCCACCGATCTTATGCAGACAGCCACTCCTCTTAACTCTTCGGAAAGCGGTGGCGCAAGCAACTCCGGGGAGGGTAGTGAACAGGAGGCGATTTACGAGAAGCTTCGGCTGTTAAATACTCAGCACGCTGCAGGACCAGGACCACTGGAGCCTGCCAGAGCAGCGCCCTTGGTAGGTCAATCACCCAACCACCTAGGAACCCGATCCAGCCACCCCCAGCTGGTGCACGGTAACCATCAGGCACTACAGCAGCATCAACAGCAGAGCTGGCCGCCCCGTCACTATTCCGGATCTTGGTACCCCACCTCTCTTAGCGAAATACCCATCTCATCGGCTCCCAATATCGCATCCGTTACGGCGTATGCATCAGGACCTTCACTTGCTCACTCACTGAGTCCACCCAACGACATCGAAAGCCTGGCCAGTATCGGTCACCAGAGAAACTGCCCCGTTGCAACGGAGGACATACATTTAAAAAAAGAACTTGATGGTCATCAGTCCGATGAAACGGGCTCCGGTGAAGGTGAAAACTCCAATGGTGGCGCTTCAAATATAGGAAACACTGAGGATGATCAAGCTCGGCTCATACTAAAAAGAAAGTTGCAACGCAATCGAACATCTTTCACGAACGACCAGATAGACAGTCTTGAAAAAGAGTTTGAACGAACACACTATCCAGATGTTTTTGCCCGCGAACGTTTGGCTGGAAAGATTGGGTTGCCAGAGGCAAGAATTCAGGTTTGGTTCTCAAACCGTCGAGCAAAATGGCGTCGCGAGGAGAAGCTGCGAAACCAGCGAAGAACACCAAATTCCACAGGAGCTAGTGCAACTTCTTCCTCTACATCGGCAACCGCCTCTTTGACTGACAGCCCTAACAGCCTAAGTGCTTGTTCCTCGCTGCTGTCCGGATCAGCTGGGGGTCCCTCAGTCAGTACCATTAATGGCTTATCGTCTCCAAGCACATTGTCTACTAATGTCAATGCTCCAACGCTTGGCGCTGGGATCGATAGCTCTGAAAGCCCAACACCAATCCCGCACATTCGGCCTAGCTGCACCTCTGACAATGACAATGGTCGTCAAAGTGAAGATTGCAGAAGAGTTTGTTCTCCATGCCCACTTGGCGTTGGCGGGCATCAAAATACTCATCATATCCAGAGCAATGGTCACGCCCAAGGTCATGCACTTGTTCCTGCCATTTCGCCACGACTCAATTTTAATAGTGGTAGTTTCGGCGCGATGTACTCCAACATGCATCATACGGCGTTATCCATGAGCGATTCATATGGGGCGGTTACGCCGATTCCGAGCTTTAACCACTCAGCTGTCGGTCCGCTGGCTCCGCCATCGCCAATACCGCAACAGGGCGATCTTACCCCTTCCTCGTTATATCCGTGCCACATGACCCTACGACCCCCTCCGATGGCTCCCGCTCACCATCACATCGTGCCGGGTGACGGTGGCAGACCTGCGGGCGTTGGCCTAGGCAGTGGCCAATCTGCGAATTTGGGAGCAAGCTGCAGCGGATCGGGATACGAAGTGCTATCTGCCTACGCGTTGCCACCGCCCCCTATGGCGTCGAGCTCTGCTGCTGATTCAAGCTTCTCAGCCGCGTCCAGTGCCAGCGCTAATGTGACCCCACATCACACCATAGCCCAAGAATCATGCCCCTCTCCGTGTTCAAGCGCGAGCCACTTTGGAGTTGCTCACAGTTCTGGGTTTTCGTCCGACCCGATTTCACCGGCTGTATCTTCGTATGCACATATGAGCTACAATTACGCGTCGTCCGCTAACACCATGACGCCTTCCTCCGCCAGCGGCACATCAGCACACGTGGCCCCGGGAAAACAACAGTTCTTCGCCTCCTGTTTCTACTCACCGTGGGTCTAGCTCGAGGGTACC |

| 850-Ey-1m2d3d (808 bp) |
| --- |
| TTAGGCGCGCCCATTAACTCGAGTCTGGTTTCCGATTCCGATTTCGCTTCCTCCTGCCAACTTATTTCTATATCTTCTCCCTATGTGGCTTGTGTGTGTGAAACAAAAACGTTTGTTTCAATACGTTGGCTTCGTGCATTTTACGGTGTTGGGAAACAGACGAAATGGACTCATAATTGATGTTAAGTGTCTGCCACAGTCGCAGCCGCAAATTCAGTGGCACAACTCCGTCGCAGCCAAATGCCATTTGCTTTTCACATCCAGGTCGAACGGCGTTGCCTTGTTGACTTTGTTTTTGCTACTCATTGCCGCGATTTGGGTTAGGCATGGGGTATGTGCGCACTGTGGGAACTTTGGATTACTCAGATGAAACAGCATTTAGGACACTATGCAGCTGGAAAGATAAACTAGTTGATAGCTACTCATTTACTCATTTACTACTTACTACTAATTTAATGCATTTTTAACAACTTTAAGCTACACAAGCCAAAACTAATGGGTATTTTATAGTCCTATTTAACCCCTTTAACGAATGCATCCTTTTACCTTTTTTGGTCACGGCAGCTGAACTCTGCCCTTTCGTTGGGGGTGACTCCTCCCTCCCGTACTCCCTCCCTCCCTCCCCTCCCTCTCCGCGCCACAGTCGACCTTGTCAAGTACCTTGTTAGCTGTTGGGCAAATGCAGCGGGATCGAAAATAAAAAGCGAAACGCATCGAGAACTTCCCAAGAAAACGGCGAGTCAAAGTTGAGAAAACGCTGCTTCCGTTTAATTGACAATTGAACCCGAACCCGGACCGAACTCCTGGAGAATATGTACGGCGGCCGCATATAT |
| 850-SuH-1m2m3m6m (850 bp) |
| TTAGGCGCGCCCATTAACTCGAGTCTGGTTTCCGATTCCGATTTCGCTTCCTCCTGCCAACTTATTTCTATATCTTCTCCCTTTGTGCCCTGTGTGTGTGCGACAGATACGTTTGTTTCAATACGTTGGCTTCGTGCATTTTACGGTGTACGGATACAGACGAAATGGACTCATTGATTCCAATTGACTGATTTCAATTGATGTTAAGTGTCTGCCACAGTCGCAGCCGCAAATTCAGTGGCACAACTCCGTCGCAGCCAAATGCCATTTGCTTTTCACATCCAGGTCGAACGGCGTTGCCTTGTTGACTTTGTTTTTGCTACTCATTGCCGCGATTTGGGTTAGGCATGGGGTATGTGCGCACTGTCGGATCTTTGGATTACTCAGATGAAACAGCATTTAGGACACTATGCAGCTGGAAAGATAAACTAGTTGATAGCTACTCATTTACTCATTTACTACTTACTACTAATTTAATGCATTTTTAACAACTTTAAGCTACACAAGCCAAAACTAATGGGTATTTTATAGTCCTATTTAACCCCTTTAACGAATGCATCCTTTTACCTTTTTTGGTCACGGCAGCTGAACTCTGCCCTTTCGTTGGGGGTGACTCCTCCCTCCCGTACTCCCTCCCTCCCTCCCCTCCCTCTCCGCGCCACAGTCGACCTTGTCAAGTACCTTGTTAGCTGTTGGGCAAATGTGCCACACAAGTGGCTCACATCAGCGGGATCGAAAATAAAAAGCGAAACGCATCGAGAACTTCCCAAGAAAACGGCGAGTCAAAGTTCAGATAACGCTGCTTCCGTTTAATTGACAATTGAACCCGAACCCGGACCGAACTCCTGGAGAATATGTACGGCGGCCGCATATAT |
| 850-SuH-1-6m (850 bp) |
| TTAGGCGCGCCCATTAACTCGAGTCTGGTTTCCGATTCCGATTTCGCTTCCTCCTGCCAACTTATTTCTATATCTTCTCCCTTTGTGCCCTGTGTGTGTGCGACAGATACGTTTGTTTCAATACGTTGGCTTCGTGCATTTTACGGTGTACGGATACAGACGAAATGGACTCATTGATTCCAATTGACTGATTTCAATTGATGTTAAGTGTCTGCCACAGTCGCAGCCGCAAATTCAGTGGCACAACTCCGTCGCAGCCAAATGCCATTTGCTTTTCACATCCAGGTCGAACGGCGTTGCCTTGTTGACTTTGTTTTTGCTACTCATTGCCGCGATTTGGGTTAGGCATGGGGTATGTGCGCACTGTCGGATCTTTGGATTACTCAGATGAAACAGCATTTAGGACACTATGCAGCTGGAAAGATAAACTAGTTGATAGCTACTCATTTACTCATTTACTACTTACTACTAATTTAATGCATTTCTAAGTACTTTAAGCTACACAAGCCAAAACTAATGGGTATTTTATAGTCCTATTTAACCCCTTTAACGAATGCATCCTTTTACCTTTTTTGGTCACGGCAGCTGAACTCTGCCCTTTCGTTGGGGGTGACTCCTCCCTCCCGTACTCCCTCCCTCCCTCCCCTCCCTCTCCGCGCCACAGTCGACCATGTCAAGTACCTTGTTAGCTGTTGGGCAAATGTGCCACACAAGTGGCTCACATCAGCGGGATCGAAAATAAAAAGCGAAACGCATCGAGAACTTCCCAAGAAAACGGCGAGTCAAAGTTCAGATAACGCTGCTTCCGTTTAATTGACAATTGAACCCGAACCCGGACCGAACTCCTGGAGAATATGTACGGCGGCCGCATATAT |
| 850-Scro-1-7d (724 bp) |
| TTAGGCGCGCCCATTAACTCGAGTCTGGTTTCCGATTCCGATTTCGCTTCCTCCTGCCAACTTATTTCTATATCTTCTCCCTTTGTGCCCTGTGTGTGTGAAACAAAAACGTTTGTTTCAATACGTTGGCTTCGTGCATTTTACGGTGTTGGGAAACAGACGAAATGGACTCATTGATTCCAATTGACTGATTTCAAAGTCGCAGCCGCAAATTCAGTGGCACAACTCCGTCGCAGCCAAATGCCATTTGCTTTTCACATCCAGGTCGAACGGCGTTGCCTTGTTGACTTTGTTTTTGCTACTCATTGCCGCGATTTGGGTTAGGCATGGGGTATGTGCGCACTGTGGGAACTTTGGATTACTCAGATGACAGCTGGAAAGATAAACTAGTTGATAGCTACTCATTTACTCATTTACTACTTACTACTAATTTAATGCATTTTTAACAACTTTAAGCTACATTTATAGTATGCATCCTTTTACCTTTTTTGGTCACGGCAGCTGAACTCTGCCCTTTCGTTGGGGGTGACTCCTCCCTCCCGTACTCCCTCCCTCCCTCCCCTCCCTCTCCGCGCCACAGTCGACCGTTGGGCAAATGTGCCACACAAGTGGCTCACATCAGCGGGATCGAAAATAAAAAGCGAAACGCATCGAGAACTTCCCAAGAAAACGGCGAGTCAAAGTTGAGAAAACGCTGCTTCCGAACCCGGACCGAACTCCTGGAGAATATGTACGGCGGCCGCATATAT |
| 850-Slp1-1d (829 bp) |
| TTAGGCGCGCCCATTAACTCGAGTCTGGTTTCCGATTCCGATTTCGCTTCCTCCTGCCAACTTATTTCTATATCTTCTCCCTTTGTGCCCTGTGTGTGTGAAACAAAAACGTTTGTTTCAATACGTTGGCTTCGTGCATTTTACGGTGTTGGGAAACAGACGAAATGGACTCATTGATTCCAATTGACTGATTTCAATTGATGTTAAGTGTCTGCCACAGTCGCAGCCGCAAATTCAGTGGCACAACTCCGTCGCAGCCAAATGCCATTTGCTTTTCACATCCAGGTCGAACGGCGTTGCCTTGTTTGCCGCGATTTGGGTTAGGCATGGGGTATGTGCGCACTGTGGGAACTTTGGATTACTCAGATGAAACAGCATTTAGGACACTATGCAGCTGGAAAGATAAACTAGTTGATAGCTACTCATTTACTCATTTACTACTTACTACTAATTTAATGCATTTTTAACAACTTTAAGCTACACAAGCCAAAACTAATGGGTATTTTATAGTCCTATTTAACCCCTTTAACGAATGCATCCTTTTACCTTTTTTGGTCACGGCAGCTGAACTCTGCCCTTTCGTTGGGGGTGActcctccctcccgtactccctccctccctcccctccctctccGCGCCACAGTCGACCTTGTCAAGTACCTTGTTAGCTGTTGGGCAAATGTGCCACACAAGTGGCTCACATCAGCGGGATCGAAAATAAAAAGCGAAACGCATCGAGAACTTCCCAAGAAAACGGCGAGTCAAAGTTGAGAAAACGCTGCTTCCGTTTAATTGACAATTGAACCCGAACCCGGACCGAACTCCTGGAGAATATGTACGGCGGCCGCATATAT |
